# Une embolie pulmonaire simulant un syndrome coronarien aigu

**DOI:** 10.11604/pamj.2019.33.75.18355

**Published:** 2019-06-03

**Authors:** Fadoua Mouedder, Houssam Laachach, Abdelmalek Elyandouzi, Alaa Fliti, Chaymae Toutai, Nabila Ismaili, Noha Elouafi

**Affiliations:** 1 Service de Cardiologie, CHU Mohammed VI, Faculté de Médecine, Université Mohammed Premier, Oujda, Maroc

**Keywords:** Embolie pulmonaire, syndrome coronaire aigu, troponine, Pulmonary embolism, severe coronary syndrome, troponin

## Abstract

L'embolie pulmonaire est affection médicale aiguë et grave. Sa présentation clinique n'est pas pathognomonique et peut simuler d'autres urgences médico-chirurgicales. Nous rapportons le cas d'un patient admis dans un tableau de syndrome coronarien aigu avec modification électrique et ascension des enzymes cardiaques sans substrat significatif à la coronarographie, faisant diagnostiquer une embolie pulmonaire.

## Abstract (en)

Pulmonary embolism is an acute and severe medical condition. Its clinical characteristics are not pathognomonic and can mimick other medico-surgical emergencies. We report the case of a patient admitted with a clinical manifestation of acute coronary syndrome with electrical changes and elevation in cardiac enzymes without abnormal substrate on the coronarography, enabling diagnosis of pulmonary embolism.

## Introduction

Manifestation potentiellement grave de la maladie thromboembolique, l'embolie pulmonaire est caractérisée par une variabilité de sa présentation clinique. L'orientation des examens complémentaires indispensables au diagnostic de certitude est basée sur des scores de probabilité clinique mais aussi sur un bon sens de clinicien. L'embolie pulmonaire est décrite comme étant la grande simulatrice de la pathologie coronaire aiguë, bien que les signes électrocardiographiques soient classiques, elle peut s'accompagner d'anomalies faisant évoquer à tort un infarctus du myocarde.

## Patient et observation

**Figure 1:**
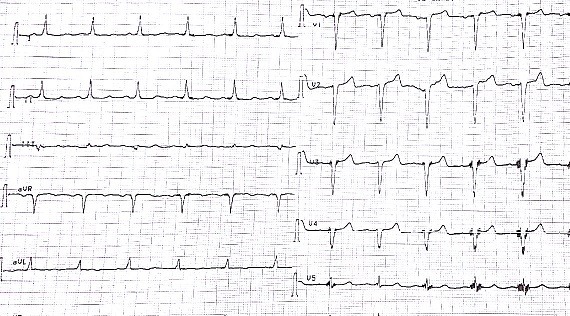
ECG à l'admission du malade

**Figure 2:**
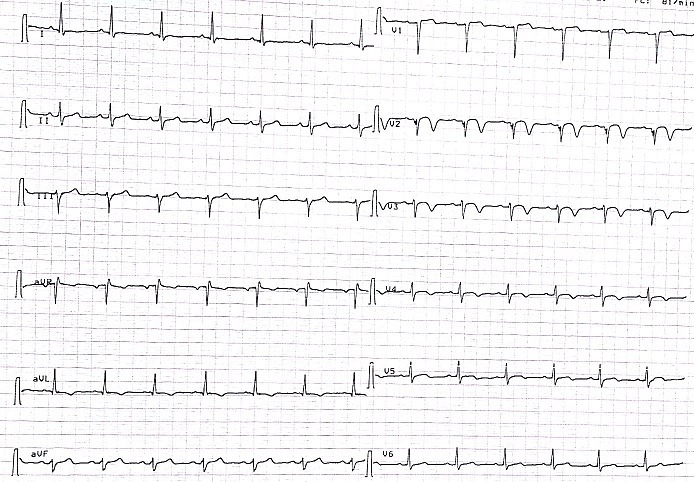
ECG objectivant des modifications électriques

**Figure 3:**
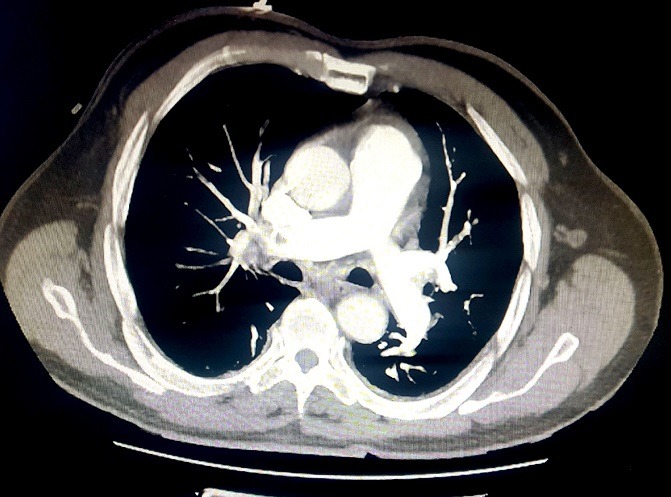
Coupe angio-TDM mettant en évidence une embolie pulmonaire

Nous rapportons le cas d'un patient âgé de 73 ans, diabétique depuis 12 ans équilibré sous antidiabétiques oraux et hypertendu connu depuis 2 ans sous Losartan 50 mg qui était admis dans notre service après 10 heures d'apparition d'une douleur thoracique angineuse aiguë, intense, permanente, non irradiante et associée à des sueurs et une lipothymie. L'examen clinique à l'admission trouvait: une pression artérielle à 125/80 mmHg, une fréquence cardiaque à 98 battements par minute, une température à 37,5°C et une fréquence respiratoire à 15 cycles par minute avec une saturation périphérique à l'air ambiant à 96%. L'examen somatique était strictement normal, notamment pas de signes cliniques de thrombose veineuse des membres inférieurs et pas de frottement péricardique ni d'asymétrie tensionnelle. L'électrocardiogramme initialement réalisé (Figure 1) a objectivé un rythme régulier sinusal, axe du cœur normal, PR à 120 ms, onde Q en DIII. Le patient a fait apparaitre quelques heures après un ST suspendu en antéro septal (Figure 2). La radiographie thoracique a été sans particularité. La C-reactive protein était à 19 mg/L et le taux de troponine à 233 (normale < 26ng/L). Le reste du bilan biologique était normal, notamment une fonction rénale correcte. L'ETT limitée par la mauvaise echogénité a objectivé une bonne fonction systolo-diastolique bi ventriculaire, sans épanchement péricardique ni dilatation aortique initiale. Devant ce tableau de douleur thoracique aiguë chez un patient ayant de multiples facteurs de risque cardiovasculaire avec une modification électrique et une cinétique de troponine positive: le diagnostic d'un syndrome coronarien aigu a été posé et traité comme tel. La coronarographie objectivant une sténose à 60% de la bifurcation IVA-première diagonale d'allure chronique, relevant d'un traitement médical. Le deuxième jour d'hospitalisation, le patient est devenu légèrement dyspnéique avec une Sa 02 à l'air ambiant à 89% sans signes physiques d'insuffisance cardiaque ni de modification électrique. Le taux de dimères et des pro BNP était élevée. L'angioscanner thoracique a objectivé un aspect en faveur d'une embolie pulmonaire périphérique bilatérale; sans atteinte parenchymateuse, avec une lame de pleurésie bilatérale (Figure 3). L'embolie pulmonaire a été stratifiée à bas risque avec score de PESI à 0. Un écho-Doppler veineux des membres inférieurs a été fait dans le cadre du bilan étiologique montrant un aspect en faveur d'une thrombose veineuse profonde du membre inférieur gauche. Le reste du bilan étiologique est revenu en faveur d'un adénocarcinome prostatique chirurgical. L'évolution clinique initiale sous anticoagulation était favorable, puis le patient fut adressé en urologie pour complément de prise en charge.

## Discussion

L'embolie pulmonaire est une pathologie fréquente et potentiellement grave, nécessitant une prise en charge urgente et adéquate. Le diagnostic d'une embolie pulmonaire n'est pas toujours aisé, malgré le recours à des scores de probabilité clinique, comme le score de Wells [1] et de Genève [2], permettant de la classer en une probabilité faible, intermédiaire ou forte et d'orienter par la suite les examens complémentaires. L'angioscanner thoracique est l'examen de référence dans le diagnostic de l'embolie pulmonaire. De nombreuses études ont montré la sécurité d'exclure l'EP sur la base d'un résultat négatif, en cas de probabilité clinique non forte mais des D-dimères positifs ou en cas de probabilité clinique forte [3,4]. En parallèle avec l'amélioration de la technologie de l'imagerie par médecine nucléaire, la scintigraphie tomographique de ventilation/perfusion (V/P), permet actuellement l'acquisition d'images tridimensionnelles en une meilleure résolution et visualisation des défauts de perfusion de petites tailles, ce qui augmente les performances diagnostiques par rapport à la scintigraphie (V/P) classique [5]. L'échocardiographie n'est pas recommandée en première intention dans le diagnostic de l'EP vue sa faible sensibilité qui est l'ordre de 60 à 70%. L'ETT trouve sa place dans le diagnostic des formes graves, en particulier lorsque le patient est trop instable pour réaliser un angioscanner thoracique ou qu'il existe un état de choc cardiogénique ou des signes d'insuffisance cardiaque droite [6]. Le score PESI (Pulmonary Embolism Severity Index [7] et sa version simplifiée (sPESI), permettent d'identifier avec au moins autant d'exactitude les patients à bas risque que la combinaison de paramètres échographiques et biologiques.

Les symptômes cliniques comme la dyspnée, la douleur thoracique et la lipothymie sont peu spécifiques, pouvant aussi être rapportés au cours des syndromes coronariens aigus, de la dissection aortique, des pneumothorax et des péricardites; Posant ainsi le problème de diagnostic différentiel [8]. L'élévation des marqueurs biologiques tel que la troponine et les pro BNP, peut être retrouvée aussi bien dans l'embolie pulmonaire que dans les syndromes coronariens [9,10]. Les D-dimères qui s'avèrent utiles dans l'exclusion de l'embolie pulmonaire, en cas de probabilité clinique faible ou intermédiaire selon les recommandations européennes de l'embolie pulmonaire, peuvent également être élevés de façon modérée dans les syndromes coronariens et la dissection aortique [11]. Pour ce qui est des anomalies électriques accompagnants l'embolie pulmonaire, elles ont été bien décrites depuis des décennies dans la littérature [12] sous forme de cas isolés ou de courtes séries où le diagnostic différentiel embolie pulmonaire vs coronaropathie aiguë a été soulevé. Leur sensibilité et leur spécificité ne sont pas de pointe. Le classique aspect S1Q3T3, est le premier signe qui a été décrit par Mc Ginn 1935 [13], avec une spécificité élevée mais une faible sensibilité pour le diagnostic de l'embolie pulmonaire. La tachycardie sinusale et le bloc de branche droit incomplet sont les plus fréquemment rencontrés chez les patients atteints d'une embolie pulmonaire, dans l'étude menée par Rodger *et al.* portant sur 246 cas [14]. Une autre étude de 190 patients menée par Sukhia *et al*. a conclu que l'association d'au moins deux des cinq critères suivants (S1, Q3, S1Q3, tachycardie sinusale et tachyarythmie supraventriculaire) a une sensibilité de 78 % et une spécificité de 96 % pour le diagnostic d'embolie pulmonaire si le contexte clinique en oriente [15]. Une inversion de l'onde T a également été décrite dans le territoire antérieur et inférieur [16,17].

La modification du segment ST à la phase aigüe d'une embolie pulmonaire reste un signe rare, seulement quelques cas ont été décrits [18-20]. Dans notre cas la modification électrique a intéressé le territoire antéro septal. L'explication physiopathologique de ce phénomène est actuellement inconnue, et des hypothèses ont été avancées dans ce sens [18]: 1) une ischémie mécanique (compression coronaire par dilatation ventricule droit); 2) un spasme coronaire secondaire à l'hypoxie; 3) une embolie paradoxale au niveau coronaire; 4) diminution aiguë de la précharge ventriculaire gauche. Le syndrome coronarien aigu est une urgence à la fois diagnostique et thérapeutique, la prise en charge est une course contre la montre « time is muscl » raison pour laquelle le traitement médicamenteux associé souvent à un traitement interventionnel est rapidement instauré. Ce traitement comportant entre autres une dose curative des anticoagulants constituent par principe la base de traitement de l'embolie pulmonaire non sévère. Notre cas, qui rejoint les cas décrits dans la littérature mondiale [17-21], confirme que l'embolie pulmonaire peut être bel et bien la grande simulatrice de la pathologie thoracique, la présence d'une modification du segment ST nous a orienté à tort vers une coronaropathie aiguë.

## Conclusion

Les signes cliniques et la symptomatologie fonctionnelle de l'embolie pulmonaire (EP) étant peu sensibles et peu spécifiques, simulent souvent d'autres pathologies thoraciques aiguës notamment un syndrome coronarien. Les marqueurs biologiques, l'ETT et l'ECG peuvent parfois porter confusion. Le choix d'autres explorations paracliniques est bien guidé par des scores cliniques ainsi que par un bon sens de clinicien.

## Conflits d’intérêts

Les auteurs ne déclarent aucun conflit d’intérêts.
